# Powder–Liquid Versus Premixed Putty MTA from the Same Manufacturer for Full Pulpotomy in Permanent Molars: A Short-Term Non-Randomized Observational Cohort Study

**DOI:** 10.3390/jcm15197563

**Published:** 2026-09-29

**Authors:** Enes Bardakçı, Mehmet Ali Arslanparçası

**Affiliations:** 1 Department of Pediatric Dentistry, Faculty of Dentistry, Harran University, Şanlıurfa 63300, Türkiye; 2 Dental Prosthetics Technology Program, Vocational School of Health Services, Harran University, Şanlıurfa 63300, Türkiye; arslanparcasi@harran.edu.tr

**Keywords:** mineral trioxide aggregate, pulpotomy, vital pulp therapy, permanent molar, calcium silicate, reversible pulpitis

## Abstract

**Background/Objectives:** Premixed calcium silicate putties are used in vital pulp therapy, but direct comparisons with powder–liquid MTA from the same manufacturer remain scarce. This exploratory study compared the short-term combined clinical and radiographic success of the two formulations in permanent molars treated with full pulpotomy for a recorded diagnosis of reversible pulpitis, and the association between cavity surface group and outcome. **Methods:** In this single-center, non-randomized observational cohort study with material allocation concealed during outcome assessment, treatment records (June–December 2025; patients aged 6–15 years) were screened, and eligible patients were recalled for standardized assessment (June–July 2026). Of 82 eligible patients, 60 (30 per group) completed recall; completion was 63.8% with powder–liquid MTA and 85.7% with putty. Two examiners assessed radiographs independently. The primary outcome was combined clinical and radiographic success; missing outcomes were examined in a sensitivity analysis. **Results:** Median follow-up was 11 months. Among recalled patients, combined success was 90.0% (27/30) with powder–liquid MTA and 93.3% (28/30) with putty (difference 3.3 percentage points; 95% CI, −12.7 to 19.7; *p* = 1.000). Imputing all missing outcomes as failures gave 57.4% and 80.0%, respectively. Success was 100% in single-surface and 88.6% in multi-surface cavities (*p* = 0.311). **Conclusions:** In this small, single-center, non-randomized cohort, both formulations showed high short-term success among patients who completed recall. Wide confidence intervals and differential loss to follow-up preclude conclusions about comparative effectiveness or equivalence; larger randomized trials with longer follow-up are required.

## 1. Introduction

The progression of deep dentine caries into the pulp tissue and the resulting pulp exposure represent a clinical situation that complicates treatment decisions, particularly in young permanent teeth. Although root canal treatment has traditionally been considered for permanent teeth diagnosed with irreversible pulpitis, clinical pulpal diagnosis does not always accurately reflect the histopathological extent of inflammation, and inflammation may remain confined to the coronal pulp in some cases [1]. These findings have supported treatment approaches based on the preservation of healthy radicular pulp, or radicular pulp with healing potential, in appropriately selected cases. Vital pulp therapies have therefore gained importance as minimally invasive treatment options aimed at maintaining pulp vitality and biological function [2].

Vital pulp therapies comprise indirect and direct pulp capping as well as partial and full pulpotomy. In full pulpotomy, the coronal pulp tissue is removed to the level of the canal orifices, and the remaining vital radicular pulp is covered with a biocompatible material. Recent clinical studies and contemporary evidence-based guidelines support full pulpotomy, performed with appropriate case selection and standardized clinical protocols, as a treatment option that can achieve high clinical and radiographic success rates in young and mature permanent teeth with carious pulp exposure [3,4,5].

Calcium hydroxide was used for many years as the standard capping material in vital pulp therapy. However, its dissolution over time, limited sealing ability, and the tunnel defects observed in the hard tissue barrier have prompted the development of alternative materials. A large practice-based randomized clinical trial reported a lower 24-month failure probability for mineral trioxide aggregate (MTA) than for calcium hydroxide after direct pulp capping of permanent teeth [6]. Owing to its biocompatibility, ability to set in a moist environment, sealing ability, and support of mineralized tissue formation, MTA has become a widely used hydraulic calcium silicate-based material in vital pulp therapy [7].

Conventional MTA materials are prepared before application by mixing the powder and liquid according to the ratio specified by the manufacturer. Variations in the powder-to-liquid ratio have been shown to affect the solubility and porosity of the set material, with higher liquid proportions increasing both [8]. The need for mixing, prolonged setting time, and difficulties in handling may also limit the clinical use of conventional MTA. To overcome some of these limitations, premixed calcium silicate-based putty materials that can be applied directly without additional mixing have been developed. Their standardized consistency and ready-to-use presentation provide a practical alternative to powder–liquid systems.

The ease of use of premixed MTA putty materials does not necessarily indicate superior biological or clinical performance compared with powder–liquid systems. Studies of full pulpotomy in mature permanent teeth have reported high success rates with conventional MTA, premixed calcium silicate materials, and other calcium silicate-based cements, with no significant differences between the compared materials [4,9]. However, most of these comparisons involved products from different manufacturers, so any observed difference may reflect differences in composition, particle characteristics, radiopacifier, or additives rather than the formulation itself. Comparing a powder–liquid and a premixed putty product from the same manufacturer reduces this manufacturer-level heterogeneity, although the two formulations may still differ chemically and physically. To our knowledge, no clinical study has compared such a pair of products in permanent teeth. Moreover, substantial heterogeneity exists among studies in terms of patient age, initial pulp diagnosis, stage of root development, type of vital pulp therapy, and follow-up duration [10].

The outcome of vital pulp therapy does not depend on the pulpotomy material alone. Initial pulp diagnosis, aseptic working conditions, hemorrhage control, preservation of sound tooth structure, and the quality of the immediate coronal restoration are among the factors that may influence treatment outcome [11]. The number of cavity surfaces is a potential clinical variable reflecting both the extent of coronal tooth structure loss and the complexity of the subsequent restoration. Nevertheless, a study comparing permanent molars with occlusal and proximal caries reported that caries location had no significant effect on full pulpotomy success [12]. Clinical evidence specifically evaluating cavity surface involvement as single-surface versus multi-surface in permanent teeth of children remains limited.

The primary aim of this exploratory observational study was therefore to compare the short-term combined clinical and radiographic success of powder–liquid MTA and premixed MTA putty from the same manufacturer in permanent molars treated with full pulpotomy for a recorded diagnosis of reversible pulpitis. The secondary, exploratory aim was to describe the association between cavity surface group (single-surface versus multi-surface) and combined success. Because material allocation was not randomized and the number of outcome events was expected to be small, the study was designed to provide preliminary estimates with confidence intervals rather than to test comparative effectiveness.

The null hypotheses were as follows:

- H_01_: There is no difference in combined clinical and radiographic success between the powder–liquid MTA and premixed MTA putty groups.
- H_02_: There is no difference in combined clinical and radiographic success between the single-surface and multi-surface cavity groups.

## 2. Materials and Methods

### 2.1. Study Design and Ethical Approval

This was a single-center observational cohort study combining a retrospective review of treatment records with a prospective, standardized recall assessment with material allocation concealed from the outcome assessors. Material allocation had occurred during routine clinical care and was not randomized; the primary material-group comparison and the secondary cavity-surface comparison were treated as exploratory. Treatment records were reviewed according to predefined inclusion and exclusion criteria, and current clinical and radiographic assessments of eligible patients were performed using a standardized protocol. No new pulpotomy treatment was performed within the scope of the study, and no previous treatment decision was modified for research purposes.

The study was conducted in accordance with the principles of the Declaration of Helsinki, and the research protocol was approved by the Clinical Research Ethics Committee of Harran University on 11 May 2026 (protocol no. HÜ-KAEK-2026-00130). Before the recall assessment, written informed consent was obtained from the parents or legal guardians of all participants, and assent was obtained from children of appropriate age and developmental level. The study is reported in accordance with the STROBE statement for observational studies (File S1). The study was not prospectively registered in a clinical study registry.

### 2.2. Study Population and Eligibility Assessment

The study population consisted of records of patients who underwent full pulpotomy of permanent molars between 1 June and 1 December 2025. Standardized clinical and radiographic recall assessments were performed between 15 June and 20 July 2026, after ethics committee approval. Individual follow-up duration was calculated as the interval between the treatment date and the recall assessment date.

All records from the specified treatment period were reviewed according to predefined inclusion and exclusion criteria. Eligibility was determined from the records available for the treatment period; attendance at the recall appointment was not considered an eligibility criterion.

Eligible records were categorized according to the pulpotomy material used: powder–liquid MTA or premixed MTA putty. A separate screening log of records examined and excluded before eligibility was established was not kept during the record review; the number of records screened and the reasons for exclusion before the eligibility stage therefore cannot be reconstructed reliably, and the patient flow is reported from the 82 records that met the eligibility criteria. Screening identified 82 eligible patients, each contributing one study tooth: 47 in the powder–liquid MTA group and 35 in the premixed MTA putty group. For each patient, the study tooth was the single permanent molar treated by the study operator and identified as the index tooth in the treatment records; the remaining dentition was not screened for additional eligible molars, so the number of patients who may have had more than one eligible tooth cannot be determined.

#### 2.2.1. Inclusion Criteria

Patients and teeth were considered eligible when all of the following criteria were met:

- Age 6–15 years at the date of treatment;
- A permanent molar with carious pulp exposure;
- A diagnosis of reversible pulpitis at the date of treatment;
- Treatment with full pulpotomy using BIOfactor MTA or BIOfactor MTA Putty;
- Treatment performed under local anesthesia and rubber dam isolation;
- The pulpotomy material and treatment procedures clearly identifiable from the patient records;
- Adequate clinical and radiographic records from the treatment period;
- A minimum of six months elapsed between pulpotomy and recall assessment.

#### 2.2.2. Exclusion Criteria

A patient or tooth was not considered eligible if any of the following were present:

- A diagnosis of irreversible pulpitis or pulp necrosis;
- Trauma-related pulp injury or pulp exposure;
- A sinus tract, abscess, pathological mobility, furcation or periapical radiolucency, or pathological root resorption before treatment;
- Radicular pulp necrosis detected during pulpotomy or failure to achieve hemorrhage control within the predefined period;
- Any endodontic treatment of the study tooth performed before the index pulpotomy (root canal treatment or extraction performed after the pulpotomy was recorded as an outcome, not as an exclusion; Section 2.11);
- Coronal tissue loss rendering the tooth unrestorable;
- A systemic disease or regular medication use considered capable of affecting pulp healing.

Non-attendance at the recall appointment, refusal to participate, or failure to complete the current clinical and radiographic assessments were not considered exclusion criteria during the initial eligibility assessment. These patients were not included in the complete-case outcome analysis and are reported separately in the study flow diagram. For all 82 eligible patients, clinical records covering the interval between the index pulpotomy and the end of the recall period were reviewed for root canal treatment, extraction, emergency attendance, or documented treatment of the study tooth elsewhere. For the 22 patients who did not complete recall, no such event was documented, and no interim clinical or radiographic assessment confirming success was recorded either; their outcomes are therefore genuinely unknown, and they were reported as lost to follow-up rather than classified as successes or failures.

### 2.3. Recall Procedure and Sample

Eligible patients were contacted in a computer-generated random order within each material group; treatment allocation had occurred previously during routine care and was not randomized (Section 2.4).

The study was designed as an exploratory observational study to provide preliminary clinical data on the performance of the two materials. No a priori sample size or power calculation was performed. A prespecified target of 30 patients and 30 teeth per material group was established, resulting in a total target sample of 60 patients and 60 teeth, based on the number of available eligible records and the feasibility of conducting the recall assessments. Only one permanent molar per patient was included.

Patients were contacted according to the randomized recall sequence until 30 patients with completed clinical and radiographic assessments were obtained in each material group; in both groups, reaching this target required contacting all eligible patients. If a patient did not attend the recall appointment, declined participation, or did not complete the required assessments, the next eligible patient in the randomized sequence within the same material group was contacted. Consequently, 60 patients and 60 permanent molars, comprising 30 treated with powder–liquid MTA and 30 treated with premixed MTA putty, were included in the final analysis.

### 2.4. Material Allocation

The pulpotomy treatments included in the study had been performed as part of routine clinical practice before the recall phase of the research. The pulpotomy material was not determined by the study protocol, and patients were not randomized to material groups at the time of treatment. The material used for each tooth was identified from the treatment records.

The reasons underlying material selection were not documented. Material choice may have been influenced by product availability at the time of treatment, tooth type, cavity extent, operator or supervisor preference, or parental preference, none of which can be evaluated retrospectively; residual confounding related to non-random material allocation therefore cannot be excluded. Both products were concurrently available throughout the treatment period, and no formal changeover from one product to the other occurred; the choice for each tooth was made by the operator during routine care. The distribution of treatment dates between the material groups is reported in Section 3.2.

### 2.5. Operator and Clinical Standardization

All pulpotomy treatments included in the study were performed according to a standardized clinical protocol by the same PhD student in pediatric dentistry, who had received theoretical and clinical training in vital pulp therapy as part of the doctoral program. Diagnosis, local anesthesia, isolation, removal of coronal pulp tissue, hemorrhage control, placement of the pulpotomy material, and restoration were performed under the supervision of the student’s academic supervisor, a pediatric dentistry specialist.

The same operator and the same supervisor were involved throughout the treatment period, and no change to the hemostasis method, material application, or coronal restoration procedure occurred during that period. The use of a single operator working under academic supervision was intended to minimize operator-related variability in the clinical procedures.

### 2.6. Diagnostic Criteria for Reversible Pulpitis

At the time of treatment, the diagnosis of reversible pulpitis was made by the treating clinician on the basis of pain history together with clinical and radiographic findings. For the present study, eligibility was confirmed using only those diagnostic features that could be verified retrospectively from the clinical records. The treatment-period record form did not contain mandatory structured present/absent fields for these features. Eligibility was therefore determined from the contemporaneous diagnosis of reversible pulpitis recorded by the treating clinician, together with the recorded pain history, clinical examination notes, and the preoperative radiograph. Teeth were excluded when any of the following was explicitly documented in the treatment-period record:

- Spontaneous or nocturnal pain;
- Lingering pain after removal of the stimulus;
- Tenderness to percussion or palpation at the treatment visit;
- Pathological mobility at the treatment visit;
- Periapical or, where applicable, furcation radiolucency, pathological root resorption, or pathological widening of the periodontal ligament space on the preoperative periapical radiograph, verified by review of the image.

Undocumented findings were handled as follows: the absence of an explicit negative entry for a single feature was not interpreted as a positive finding, and because the distinction between an explicitly documented negative finding and an undocumented feature was not captured patient by patient, the number of records with explicit negative documentation cannot be reported for each feature. Because the specific pulp sensibility tests used and the exact duration of pain after removal of the stimulus were not documented in a standardized format, these parameters could not be used to retrospectively verify the diagnosis. Accordingly, the study population is best described as permanent molars with a recorded diagnosis of reversible pulpitis at the time of treatment: the radiographic criterion was verifiable from the images, the recorded pain history and clinical findings could be checked only for explicitly recorded positive findings, and the sensibility test results and the duration of provoked pain depended entirely on the treating clinician’s diagnosis. This limitation is addressed in Section 4.

The decision to perform full pulpotomy was made during routine clinical treatment on the basis of clinical, radiographic, and intraoperative findings and was not determined during patient selection for the present study. Permanent molars with carious pulp exposure that were restorable, diagnosed with reversible pulpitis, and free of clinical or radiographic signs of infection were considered suitable for full pulpotomy. Full pulpotomy rather than a less extensive vital pulp procedure was the routine approach in the clinic for carious exposures of this type during the treatment period. The intraoperative findings that informed this choice for individual teeth, such as the size of the exposure, the appearance of the exposed pulp, or the characteristics of bleeding beyond the hemostasis criterion described below, were not systematically documented.

Following removal of the coronal pulp tissue, the presence of vital bleeding from the canal orifices and successful hemorrhage control within eight minutes were considered intraoperative criteria supporting preservation of the radicular pulp. Teeth showing radicular pulp necrosis, absence of vital bleeding from the canal orifices, or failure to achieve hemostasis within eight minutes were not considered suitable for full pulpotomy. These decision criteria were based on current approaches recommending the combined assessment of preoperative pulp diagnosis, radicular pulp vitality, and hemorrhage control in the management of carious pulp exposure in permanent teeth [2,3,4].

### 2.7. Pulpotomy Protocol

According to the patient records, all treatments were performed under local anesthesia (Lidofast, 2% lidocaine with 1:80,000 epinephrine; VEM İlaç, İstanbul, Türkiye) and rubber dam isolation (IsoDam; Four D Rubber Co. Ltd., Heanor, UK). After disinfection of the operative field, caries was removed, and pulp exposure was confirmed. The coronal pulp tissue was completely removed to the level of the canal orifices using a sterile rotary instrument (HORICO; Hopf, Ringleb & Co. GmbH & Cie., Berlin, Germany), and the pulp chamber was rinsed with sterile saline (Biofleks 0.9% sodium chloride; Osel İlaç, İstanbul, Türkiye).

Hemorrhage control was achieved by applying a cotton pellet, prepared from cotton rolls (RubyPlaton; İnci Dental, İstanbul, Türkiye) and sterilized before use, moistened with 2.5% sodium hypochlorite (NaOCl) to the pulp stumps under light pressure; the 2.5% solution was prepared by diluting a commercial 5% NaOCl solution (Microvem; Altun Sterilizasyon ve Medikal, Sakarya, Türkiye) with an equal volume of distilled water. The cotton pellet was applied in two-minute intervals and, if bleeding persisted, the procedure was repeated up to four times, with a maximum total hemostasis time of eight minutes. This eight-minute threshold was the routine full pulpotomy protocol used in the clinic during the treatment period; it is consistent with a previously published clinical protocol [13] and with contemporary vital pulp therapy protocols, in which hemostasis with NaOCl is generally achieved within approximately 5–10 min [14].

Teeth in which hemostasis could not be achieved within eight minutes, in which vital bleeding was absent from the canal orifices, or in which radicular pulp necrosis was identified were considered unsuitable for full pulpotomy and were therefore not eligible for the present study.

After hemostasis, the relevant pulpotomy material was placed directly over the radicular pulp tissue and the pulp chamber floor to a thickness of approximately 3 mm, as described in previous clinical studies of full pulpotomy [4]. Each material was handled according to the instructions of its manufacturer in use during the treatment period; the version or date of the instructions for use for either product was not recorded, and the original packaging inserts were not retained, so it cannot be verified retrospectively.

### 2.8. Study Groups and Materials

Group 1—Powder–liquid MTA: BIOfactor MTA (İmicryl Dental, Konya, Türkiye) was used. The material was prepared by mixing powder and liquid at the manufacturer-recommended ratio (the exact scoop-to-drop ratio and mixing time were not documented for individual teeth and cannot be verified retrospectively) on a glass slab with a metal spatula until a putty-like consistency was obtained, and was placed over the radicular pulp tissue after hemostasis.

Group 2—Premixed MTA putty: Ready-to-use BIOfactor MTA Putty (İmicryl Dental, Konya, Türkiye) was used. The material was taken directly from its packaging without additional mixing and placed over the radicular pulp tissue after hemostasis.

In both groups, excess fluid was removed from the pulp chamber with a sterile cotton pellet after hemostasis, and the radicular pulp surface was left slightly moist rather than dried before material placement. The pulpotomy material was condensed lightly with a moist cotton pellet; no separate waiting interval was observed, and no documented check of initial setting was performed before the light-cured glass ionomer base material Glass Liner^®^ (Willmann & Pein GmbH, WP Dental, Barmstedt, Germany) was applied over the pulpotomy material. The final coronal restoration was completed in the same session using Estelite Sigma Quick^®^ composite resin (Tokuyama Dental Corporation, Tokyo, Japan). According to the clinic’s standard protocol, the materials were intended to be applied in accordance with the manufacturers’ instructions; however, adherence to every handling step could not be verified for individual teeth. A periapical radiograph was taken after treatment using the standard paralleling technique. The steps described here reflect the clinic’s standard written protocol; mixing time, moisture condition, and the interval before liner placement were not recorded for individual teeth and could not be verified per tooth.

### 2.9. Recorded Variables

For each patient and tooth, the anonymous study code, age at treatment, sex, pulpotomy material, treated tooth number, cavity surface group, follow-up duration, and clinical, radiographic, and combined outcomes were recorded on a standardized data collection form. Tooth numbers were recorded according to the FDI two-digit notation. Teeth were also classified by jaw and as first or second molars. Cavities were categorized as single-surface (one surface) or multi-surface (two or more surfaces).

The stage of root development was not recorded as a standard variable during the treatment period. Apical status was therefore classified retrospectively from the immediate postoperative periapical radiograph, which served as the baseline for the radiographic comparison, by two examiners (E.B. and H.F.A.) who worked independently on coded images presented in random order, with material allocation concealed. An apex was classified as open when the apical foramen was wider than the adjacent canal or root length was incomplete, and as closed when root length was complete and the apical foramen was narrowed; a multi-rooted tooth was classified as open if any root had an open apex. Disagreements were resolved by consensus. Root maturity was not inferred from age or tooth identity. Patient names and identifying information were not transferred to the analysis dataset; all records and assessments were handled using anonymous study codes.

Age at treatment, sex, treated tooth number, material group, cavity surface group, and treatment date were obtained from the records for all 82 eligible patients, including the 22 who did not complete the recall assessment. These variables were compared between the two sets of patients to assess potential selection bias. For this comparison, time since treatment was calculated for all 82 patients as the interval between the treatment date and a common reference date, the end of the recall period (20 July 2026). Follow-up duration among the assessed patients, defined as the interval from treatment to the recall examination, is reported separately in Section 3.2.

### 2.10. Blinding and Recall Assessments

Clinical recall assessments were performed by a specialist academic in pediatric dentistry (E.B.), who had not performed any of the pulpotomy treatments but had supervised them clinically. The clinical examiner had no access to records indicating the pulpotomy material used. Material information was concealed before assessment, and the material groups were coded as A and B.

Radiographic assessments were performed independently by two examiners (E.B. and H.F.A.), both blinded to the pulpotomy material; H.F.A. is not an author of this study and had no involvement in the original treatments. The correspondence between the material codes and the actual materials was not disclosed until completion of the assessments. Clinical and radiographic outcomes were recorded separately for each tooth on standardized assessment forms and were subsequently combined to determine the overall treatment outcome.

### 2.11. Clinical Assessment

A standardized intraoral examination was performed at the recall appointment. The following clinical findings were assessed: spontaneous or lingering pain, tenderness to percussion or palpation, swelling or abscess, sinus tract, pathological mobility, inflammation of the surrounding soft tissues, and an isolated deep periodontal pocket associated with the treated tooth.

Clinical success was defined as the absence of all of these findings and no root canal treatment or extraction of the tooth performed for pulpal or periapical reasons between the index pulpotomy and the recall examination. Clinical failure was defined as the presence of at least one of these findings, or root canal treatment or extraction of the tooth performed for pulpal or periapical reasons between the index pulpotomy and the recall examination. Clinical status was classified from symptoms and examination findings at the recall visit, before the radiographs were read and before any referral decision was made; a referral made after the recall examination did not enter the classification. The clinical success criteria were based on those reported by Taha and Abdulkhader [3].

### 2.12. Radiographic Assessment

Radiographic assessment was performed using periapical radiographs obtained at the recall appointment with the paralleling technique and a beam-aiming device (Rinn XCP; Dentsply Sirona, Charlotte, NC, USA). For each patient, the postoperative baseline radiograph and the recall radiograph were evaluated side by side to identify radiographic changes occurring after treatment.

Radiographs were obtained using a Siger SIR-GT-02 intraoral X-ray unit (Zhuhai Siger Medical Equipment Co., Ltd., Zhuhai, Guangdong, China) operating at 60 kVp and 4 mA with an exposure time of 32 ms, and a Castellini X-VS digital intraoral sensor (Cefla S.C., Imola, Italy). Images were evaluated with the radiological imaging module of the Turcasoft HBYS dental practice-management software, version 1.1.0 (Turcasoft Yazılım, Samsun, Türkiye) on a 23.8-inch full-HD display (1920 × 1080 pixels; ASUS A5402WVA all-in-one computer, ASUSTeK Computer Inc., Taipei, Taiwan) in a dimly lit room. During image evaluation, the examiners were permitted to adjust magnification, brightness, and contrast.

The radiographic criteria assessed included new or enlarging periapical or furcation radiolucency and pathological internal or external root resorption. A new radiolucency was defined as a pathological radiolucent area that was absent on the postoperative baseline radiograph but present in the periapical region or, when applicable, the furcation area on the recall radiograph. Pathological internal or external root resorption was defined as pathological loss of root structure involving either the root canal wall or the external root surface when the baseline and recall radiographs were compared.

Radiographic success was defined as the absence of all of these pathological findings. The presence of at least one finding was classified as radiographic failure. The radiographic success criteria were based on those reported by Taha and Abdulkhader [3].

### 2.13. Inter-Examiner Reliability Assessment

All 60 recall radiographs were independently evaluated by two examiners. Both examiners were blinded to the material group and completed their assessments independently. The recall radiographs were obtained at the clinical examination visit but were read at least five days after the clinical assessment. Before reading, the images were labeled with anonymous study codes and presented to both examiners in a computer-generated random order different from the order of the clinical examinations. During reading, the examiners had access only to the coded postoperative baseline and recall radiographs; they had no access to the material allocation, the clinical examination findings, the other examiner’s classification, or the final treatment outcome. The single discrepant classification was resolved by joint review after both independent readings had been completed, and the clinical findings were not disclosed during that review. Because E.B. also performed the clinical examination, the coding, random ordering, and delay were intended to limit recall of individual patients’ clinical findings; residual recall cannot be excluded for this examiner and is acknowledged in the Discussion. H.F.A. had no access to the clinical findings at any stage. Inter-examiner agreement for the radiographic outcome was assessed using Cohen’s kappa coefficient based on the initial independent evaluations.

After completion of the independent assessments, cases with discrepant classifications were reviewed jointly by the two examiners, and the final radiographic outcome was determined by consensus.

### 2.14. Outcome Variables

The primary outcome of the study was combined clinical and radiographic success. A tooth was classified as successful only when both the clinical and radiographic success criteria were fulfilled. Failure of either the clinical or radiographic assessment resulted in classification as a combined failure. Clinical and radiographic success were also reported separately, and for each combined failure, whether the failure was clinical, radiographic, or both was recorded. Radiographic-only failures were regarded as clinically relevant because a new periapical or furcation radiolucency is consistent with periapical pathosis and possible loss of radicular pulp vitality, irrespective of symptoms, and such teeth were referred for further treatment (Section 3.5).

The secondary outcome was the association between cavity surface group (single-surface versus multi-surface) and combined clinical and radiographic success. Follow-up duration and the distribution of cavity surface groups were also examined to assess the comparability of the two material groups. The definitions of all clinical, radiographic, and combined outcomes are presented in Table 1.

### 2.15. Statistical Analysis

Statistical analyses were performed using IBM SPSS Statistics for Windows, version 27.0 (IBM Corp., Armonk, NY, USA). The unit of analysis was the tooth. Because only one tooth per patient was included, there was no within-patient clustering. Before analysis, the dataset was checked for missing values, coding errors, and duplicate patient numbers. Categorical variables, including sex, were presented as counts and percentages. Age was summarized as mean ± standard deviation and median (minimum–maximum), whereas follow-up duration was presented as median, interquartile range, and minimum–maximum values.

The distributions of age and follow-up duration within the material groups were assessed using the Shapiro–Wilk test and Q–Q plots. Because the assumption of normality was not satisfied for either variable, age and follow-up duration were compared between the powder–liquid MTA and premixed MTA putty groups using the Mann–Whitney U test.

Associations between material group and combined success, and between cavity surface group and combined success, were examined using 2 × 2 contingency tables. Because expected cell counts were low, two-sided Fisher’s exact tests were used for these comparisons. Clinical and radiographic success were also analyzed separately using two-sided Fisher’s exact tests. The distribution of cavity surface groups between material groups was compared using Pearson’s chi-square test because expected cell counts were adequate. Tooth type and jaw distributions were presented as counts and percentages, and exploratory comparisons between material groups were performed using two-sided Fisher’s exact tests. In addition, the proportions of eligible patients completing the recall assessment were compared between material groups using a two-sided Fisher’s exact test. To assess potential selection bias, the 60 analyzed patients and the 22 eligible patients who did not complete the recall assessment were compared with respect to sex, material group, tooth type, jaw, and cavity surface group using two-sided Fisher’s exact tests, and with respect to age and time since treatment (common reference date) using the Mann–Whitney U test. Apical status was compared between material groups and between successful and failed teeth using two-sided Fisher’s exact tests, and inter-examiner agreement on apical status was quantified with Cohen’s kappa.

Inter-examiner agreement for the initial independent radiographic classifications was assessed using Cohen’s kappa coefficient. The kappa analysis was performed before consensus resolution of discrepant cases.

The complete-case analysis of the 60 patients who completed recall was the primary analysis. In response to peer review, a post hoc scenario-based sensitivity analysis was conducted to examine how far the material comparison depends on the outcomes of the 22 eligible patients who did not complete recall, with all 82 eligible patients as the denominator. Missing outcomes were imputed under extreme assumptions (all successes; all failures; failures in one material group and successes in the other) and under intermediate assumptions in which 10%, 20%, 30%, and 50% of the missing outcomes in both groups were failures. These scenarios are not estimates of the true outcomes of the non-assessed patients; they illustrate the range of results compatible with the observed data, and the confidence intervals and *p*-values calculated for each scenario are conditional on the outcomes assigned in that scenario. Success rates were presented with 95% confidence intervals calculated using the Wilson score method. Absolute differences in success between groups were expressed in percentage points and reported with 95% confidence intervals calculated using the Newcombe hybrid score method. Because of the small number of failures, no multivariable regression model was fitted. All statistical tests were two-sided, and the significance level was set at *p* < 0.05.

## 3. Results

### 3.1. Patient Flow

A screening log was not kept before the eligibility stage (Section 2.2), so the flow is reported from the eligible records. Screening identified 82 eligible patients, each contributing one permanent molar: 47 in the powder–liquid MTA group and 35 in the premixed MTA putty group. Within each material group, eligible records were ordered according to a computer-generated random sequence, and patients were contacted for recall assessment according to this order.

During the recall process, 13 patients could not be reached, 4 declined to participate, and 5 did not attend a scheduled appointment. Recall assessment was therefore not completed in 22 eligible patients, including 17 in the powder–liquid MTA group and 5 in the premixed MTA putty group.

The recall completion rate was 63.8% (30/47) in the powder–liquid MTA group and 85.7% (30/35) in the premixed MTA putty group. In an exploratory comparison, this difference was statistically significant (two-sided Fisher’s exact test, *p* = 0.043).

**Figure 1:**
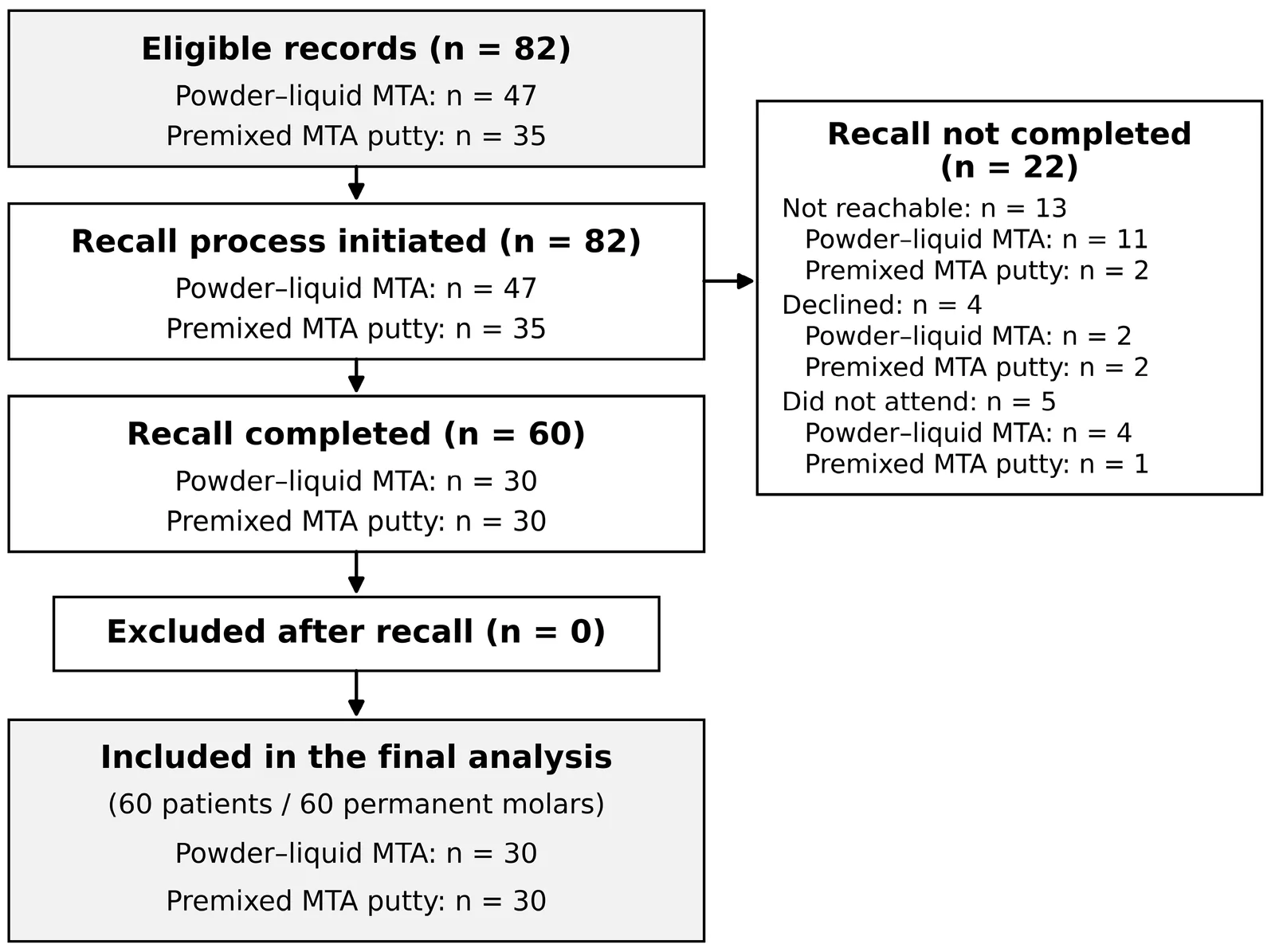
Selection of patient records and study flow. Records screened and excluded before the eligibility stage were not logged and are not shown. Of the 82 patients meeting the eligibility criteria, records were categorized according to the pulpotomy material used, and a computer-generated random recall order was applied within each material group. During the recall process, 13 patients could not be reached, 4 declined to participate, and 5 did not attend their scheduled appointment. A total of 60 patients and 60 permanent teeth (30 powder–liquid MTA and 30 premixed MTA putty) with completed clinical and radiographic recall assessments were included in the final analysis.

A total of 60 patients and 60 permanent teeth (30 powder–liquid MTA and 30 premixed MTA putty) with completed clinical and radiographic recall assessments were included in the final analysis. No patients were excluded after completion of the recall assessment, and there were no missing values in the analyzed variables. Patient selection and study flow are presented in Figure 1. No statistically significant differences were detected between the 60 analyzed patients and the 22 eligible patients who did not complete the recall assessment in age (median 11 years in both sets; 11.30 ± 2.02 versus 11.05 ± 1.68 years; Mann–Whitney U = 703.5; *p* = 0.649), sex (61.7% versus 59.1% female; *p* = 1.000), tooth type (78.3% versus 72.7% first molars; *p* = 0.571), jaw (23.3% versus 22.7% maxillary; *p* = 1.000), or cavity surface group (26.7% versus 27.3% single-surface; *p* = 1.000). Using a common reference date (20 July 2026), the median time since treatment was 11.5 months (IQR, 1.6 months) in the analyzed patients and 11.1 months (IQR, 2.0 months) in the non-assessed patients (Mann–Whitney U = 773.0; *p* = 0.239). As expected from the difference in recall completion, the two sets differed in material group distribution, the powder–liquid MTA group accounting for 50.0% of the analyzed and 77.3% of the non-assessed patients (*p* = 0.043). These comparisons concern recorded baseline characteristics only and do not exclude selection bias related to unmeasured factors, including the clinical status of the treated tooth (Supplementary Table S1). Review of the interval records identified no documented root canal treatment, extraction, emergency attendance, or treatment elsewhere for the study tooth in any of the 22 non-assessed patients, and no interim assessment confirming success; all 22 outcomes are therefore unknown.

### 3.2. Patient, Tooth, Cavity and Follow-Up Characteristics

The mean age of the 60 analyzed patients at the time of treatment was 11.30 ± 2.02 years (median, 11 years; range, 8–15 years). Of the patients, 37 (61.7%) were female, and 23 (38.3%) were male. Age did not differ significantly between the powder–liquid MTA and premixed MTA putty groups (Mann–Whitney U = 468.0; *p* = 0.794). Sex distribution also did not differ significantly between the material groups (two-sided Fisher’s exact test, *p* = 1.000).

All 60 analyzed teeth were permanent molars, of which 47 (78.3%) were first molars and 13 (21.7%) were second molars. The powder–liquid MTA group contained 26 first and 4 second molars, whereas the premixed MTA putty group contained 21 first and 9 second molars. Overall, 14 teeth (23.3%) were maxillary, and 46 (76.7%) were mandibular. Tooth type and jaw distributions did not differ significantly between the material groups (*p* = 0.209 and *p* = 0.125, respectively). The most frequently assessed teeth were the mandibular right first molar (tooth 46; n = 20) and the mandibular left first molar (tooth 36; n = 16).

Of the 60 permanent molars assessed, 16 (26.7%) had single-surface cavities, and 44 (73.3%) had multi-surface cavities. There were 7 single-surface cavities (23.3%) in the powder–liquid MTA group and 9 (30.0%) in the premixed MTA putty group. The distribution of cavity surface groups did not differ significantly between the material groups (χ^2^(1) = 0.341; *p* = 0.559). Both products were used every month from June through October 2025; the single treatment performed in November 2025 used powder–liquid MTA. No statistically significant difference in the distribution of treatment dates was detected between the material groups (Mann–Whitney U test, *p* = 0.506). Apical status could be assessed in all 60 teeth: 10 (16.7%) had open apices, five in each material group (16.7% versus 16.7%; *p* = 1.000). The two examiners agreed on apical status in 55 of the 60 teeth (91.7%; κ = 0.689; 95% CI, 0.43 to 0.94), indicating substantial agreement. Patients with open apices were younger than those with closed apices (median age 8.5 versus 12 years).

Follow-up duration ranged from 8 to 12 months. In the overall sample, the median follow-up duration was 11 months (IQR, 2 months). Follow-up was 8 months in 3 cases (5.0%), 9 months in 4 (6.7%), 10 months in 11 (18.3%), 11 months in 20 (33.3%), and 12 months in 22 (36.7%).

The median follow-up duration was 11 months (IQR, 2 months) in both material groups, with no significant difference between them (Mann–Whitney U = 442.5; Z = −0.116; *p* = 0.907). Patient, tooth, cavity, and follow-up characteristics are presented in Table 2.

### 3.3. Combined Clinical and Radiographic Success

Of the 60 teeth, 55 met the combined clinical and radiographic success criteria (91.7%; 95% CI: 81.9–96.4), whereas 5 teeth (8.3%) were classified as combined failures. In the powder–liquid MTA group, 27 of 30 teeth were successful (90.0%; 95% CI: 74.4–96.5) and 3 (10.0%) failed. In the premixed MTA putty group, 28 of 30 teeth were successful (93.3%; 95% CI: 78.7–98.2) and 2 (6.7%) failed. Combined outcomes by material group are presented in Table 3.

Because expected cell counts were below 5, a two-sided Fisher’s exact test was used to compare the material groups. No statistically significant difference was detected between the groups (*p* = 1.000). The absolute difference in success between the premixed MTA putty and powder–liquid MTA groups was +3.3 percentage points (95% CI: −12.7 to +19.7). Because the confidence interval included zero, the data did not demonstrate a statistically significant difference in combined success between the two materials.

### 3.4. Clinical and Radiographic Success as Separate Outcomes

Clinical success was 93.3% (28/30) in the powder–liquid MTA group and 100.0% (30/30) in the premixed MTA putty group (*p* = 0.492). Radiographic success was 90.0% (27/30) and 93.3% (28/30), respectively (*p* = 1.000). Of the five combined failures, none failed on clinical criteria only, three failed on radiographic criteria only, and two failed on both clinical and radiographic criteria. Clinical status was classified from symptoms and examination findings before the radiographs were read; none of the 60 teeth had undergone root canal treatment or extraction between the index pulpotomy and the recall examination, and referral for further treatment after the examination was not part of the classification. Clinical and radiographic outcomes are presented separately in Table 4.

### 3.5. Characteristics of the Failed Cases

The five teeth classified as combined failures are described individually in Table 5. All five failures were first detected at the research recall examination, after 9, 11, 8, 12, and 12 months, respectively; none had been recorded as a failure in the interval records. Coronal restoration integrity was not recorded as a standardized variable, so marginal deterioration, fracture, or leakage cannot be reported for individual teeth. One of the five failed teeth (case 2) had an open apex, and four had closed apices; combined success was 90.0% (9/10) in teeth with open apices and 92.0% (46/50) in teeth with closed apices (*p* = 1.000). Where a failure was first detected at recall, the interval given in Table 5 is the time from pulpotomy to the recall examination, not the time of onset of failure, which could not be determined. All five had multi-surface cavities, and in each case the radiographic finding was a new periapical radiolucency; in two teeth this was accompanied by pain or tenderness to percussion. The patients and parents were informed of the findings, the treatment options were explained, and the patients were referred to the relevant clinic for further care; subsequent treatment was not followed as part of the study protocol.

### 3.6. Combined Success by Cavity Surface Group

All 16 teeth in the single-surface cavity group met the combined clinical and radiographic success criteria (100.0%; 95% CI: 80.6–100.0). In the multi-surface cavity group, 39 of 44 teeth were successful (88.6%; 95% CI: 76.0–95.0), whereas 5 teeth (11.4%) were classified as failures.

Because of low expected cell counts, a two-sided Fisher’s exact test was used to compare the cavity surface groups. Although the success rate was numerically higher in single-surface cavities, no statistically significant difference was detected between the groups (*p* = 0.311). The absolute difference in success between the single-surface and multi-surface cavity groups was +11.4 percentage points (95% CI: −9.0 to +24.0) (Table 6).

### 3.7. Inter-Examiner Reliability Results

The first examiner classified five teeth and the second examiner four teeth as radiographic failures, and the examiners agreed on four of these. Overall, the two examiners agreed on the radiographic outcome in 59 of the 60 teeth (98.3%) and disagreed in one tooth, which was resolved by consensus in favor of failure, giving five radiographic failures. Inter-examiner agreement was almost perfect (Cohen’s κ = 0.880; 95% CI, 0.65 to 1.00; *p* < 0.001).

### 3.8. Sensitivity Analysis for Missing Outcomes

Seventeen of 47 eligible patients in the powder–liquid MTA group (36.2%) and 5 of 35 in the premixed MTA putty group (14.3%) did not complete recall. Table 7 shows the combined success proportions, with all 82 eligible patients as the denominator, under the assumptions described in Section 2.15. Under the extreme assumptions, combined success ranged from 57.4% to 93.6% in the powder–liquid MTA group and from 80.0% to 94.3% in the premixed MTA putty group. The estimated difference between groups ranged from −13.6 percentage points, when the missing outcomes were assumed to favor the powder–liquid MTA group, to +36.8 percentage points under the opposite assumption, and reached +22.6 percentage points (95% CI, 2.0 to 39.9) when all missing outcomes were assumed to be failures. Both the magnitude and the direction of the difference therefore change under alternative assumptions; the comparison between materials depends materially on the outcomes of the patients who did not return, particularly in the powder–liquid MTA group. The confidence intervals and *p*-values in Table 7 are conditional on the outcomes assigned in each scenario.

## 4. Discussion

This study compared the short-term combined clinical and radiographic success of powder–liquid BIOfactor MTA and premixed BIOfactor MTA Putty from the same manufacturer in permanent molars treated with full pulpotomy. The eligible age range was 6–15 years, whereas the analyzed sample ranged from 8 to 15 years. Combined success was 90.0% in the powder–liquid MTA group, 93.3% in the premixed MTA putty group, and 91.7% overall. No statistically significant difference was detected between the material groups (*p* = 1.000). Combined success was 100.0% in single-surface cavities and 88.6% in multi-surface cavities, although this difference was also not statistically significant (*p* = 0.311). These proportions apply to the patients who completed recall. The confidence interval around the material difference (−12.7 to 19.7 percentage points) is compatible with clinically relevant differences in either direction, and the sensitivity analysis shows that both the magnitude and the direction of the difference change with the assumptions made about the patients who did not return (Section 3.8). The absence of a statistically significant difference is therefore not evidence of clinical equivalence, and the findings should be read as descriptive and exploratory rather than as a test of comparative effectiveness.

The overall combined success rate of 91.7% is consistent with current evidence indicating that full pulpotomy can achieve high success rates in permanent teeth with carious pulp exposure. A meta-analysis of 25 randomized clinical trials reported an overall success rate of 86.7% for permanent tooth pulpotomy, increasing to 92.0% among teeth diagnosed with normal pulp or reversible pulpitis. The similarity between that estimate and the present findings may be related to the inclusion of teeth diagnosed with reversible pulpitis, the absence of preoperative clinical or radiographic signs of pulpal or periapical disease, and the use of a standardized clinical protocol under rubber dam isolation [15].

The principal finding of this study was that no statistically significant difference could be demonstrated between the short-term combined success of the powder–liquid and premixed putty formulations from the same manufacturer. Direct clinical comparisons of powder–liquid and premixed putty calcium silicate materials from the same manufacturer remain limited. In a randomized clinical study comparing NeoMTA Plus and NeoPUTTY (NuSmile, Houston, TX, USA) and Biodentine (Septodont, Saint-Maur-des-Fossés, France) in primary molars, combined success at 24 months was reported as 97% for NeoMTA Plus and 92% for NeoPUTTY, with no statistically significant difference between the materials. Because primary and permanent teeth differ in pulp biology, root development, treatment indications, and failure criteria, those findings cannot be directly extrapolated to the present population. Nevertheless, the lack of clear clinical superiority of the premixed putty formulation over a powder–liquid calcium silicate material is broadly consistent with the present findings [16].

Premixed putty materials require no additional mixing, are supplied at a standardized consistency, and can be applied directly, which may facilitate clinical use, particularly in pediatric patients. However, these handling advantages do not necessarily translate into superior biological or clinical outcomes. Laboratory comparisons of premixed and conventionally mixed calcium silicate cements have demonstrated differences in composition, setting time, microhardness, radiopacity, and pH [17]. The two BIOfactor formulations evaluated here have not been compared physicochemically, so, even though they are produced by the same manufacturer, they cannot be assumed to be chemically or physically identical. The present study compared the overall clinical performance of two commercial formulations and was not designed to isolate the independent effect of material presentation or mixing method. Accordingly, the numerical difference of 3.3 percentage points in favor of the premixed putty group should not be attributed solely to its ready-to-use presentation.

In the secondary analysis, all 16 teeth with single-surface cavities were successful, whereas all five combined failures occurred in teeth with multi-surface cavities. This is an observation, not a demonstrated mechanism. Greater loss of coronal tooth structure, wider restoration margins, and proximal surface involvement could increase the risk of restoration fracture, marginal deterioration, and coronal microleakage, but restoration integrity was not assessed as a graded variable in this study, so the contribution of restorative problems cannot be separated from other possible causes of failure. A large retrospective study reported an association between an increasing number of restored surfaces and failure of vital pulp therapy [18]. However, the single-surface subgroup in the present study comprised only 16 teeth and included no failures, resulting in substantial imprecision. The 95% confidence interval for the 11.4 percentage-point difference in combined success between single-surface and multi-surface cavities included zero. The observed pattern is therefore hypothesis-generating only.

The use of the same hemostasis and restorative protocol in both material groups helped to reduce clinical variability that could influence treatment outcome. In both groups, the pulpotomy material was covered with the same light-cured glass ionomer liner and composite resin restoration, thereby standardizing the coronal restorative procedure. However, restoration integrity was not evaluated as a graded outcome variable. A five-year follow-up study of mature permanent teeth reported that both pulpal and restorative failures may occur over time [19]. Long-term evaluation of pulpotomy outcomes should therefore include graded assessment of coronal restoration integrity, including marginal integrity, fracture, and leakage, in addition to pulpal and periapical findings.

The median follow-up duration was 11 months in both material groups, with no statistically significant difference between them (*p* = 0.907), reducing the likelihood that the comparison of success rates was materially influenced by differences in follow-up duration. Nevertheless, the 8–12-month follow-up period allows assessment of short-term outcomes only. In a prospective study of young patients followed for a mean of more than 11 years, full pulpotomy achieved complete clinical and radiographic success, although late radiographic changes such as pulp canal obliteration were observed in about one-fifth of the molars [20]. A recent meta-analysis reported success rates of 96%, 90%, and 96% for MTA pulpotomy at 6, 12, and 24 months, respectively, and significantly more favorable outcomes in teeth with open apices [21]. In the present cohort, open apices were equally distributed between the material groups (five per group), so root maturity is unlikely to explain the observed material comparison; success was similar in teeth with open and closed apices, but with only ten open-apex teeth and one failure among them, the study could not assess the prognostic effect reported in the literature. Future studies should record the stage of root development prospectively and account for it in the analysis.

The strengths of this study include the performance of all treatments by the same PhD student according to a standardized clinical protocol, supervision of the treatment procedures by a specialist academic in pediatric dentistry, and the use of the same hemostasis and restorative protocols in both material groups. Clinical recall assessments were performed by an examiner blinded to the material group, and radiographic assessments were performed independently by two examiners blinded to material allocation, with the inter-examiner agreement reported from the initial readings. Including only one tooth per patient eliminated within-patient clustering. The material groups had similar median follow-up and similar cavity surface distributions; however, maxillary teeth accounted for 13.3% of the powder–liquid group and 33.3% of the putty group, and second molars for 13.3% and 30.0%, respectively. These differences are noticeable despite non-significant tests, and, together with the unrecorded reasons for material choice, they mean that residual confounding cannot be excluded. Open apices occurred in 16.7% of the teeth in each group, and both products were represented in every month from June through October 2025; however, these observations do not eliminate residual confounding. Because only five failures occurred, no adjusted analysis was attempted, and the comparison is presented as unadjusted and exploratory.

The two principal limitations of this study are the non-random allocation of materials and the differential loss to follow-up; both are major rather than incidental, and both bear directly on the material comparison. Recall assessment could not be completed in 22 of the 82 patients who met the eligibility criteria. The recall completion rate was 63.8% in the powder–liquid MTA group and 85.7% in the premixed MTA putty group, and this difference was statistically significant in the exploratory comparison. Because attendance at recall may have been influenced by patient compliance, current symptoms, access to transportation, or the family’s perception of treatment outcome, differential non-participation bias cannot be excluded. No statistically significant differences were detected between the analyzed and non-assessed patients in age, sex, tooth type, jaw, or cavity surface group, although these comparisons do not exclude selection bias: patients may fail to return because the tooth is asymptomatic, because they have already sought treatment elsewhere, or for reasons unrelated to the tooth, and baseline similarity does not establish that their outcomes were similar. Differential non-attendance is a plausible selection mechanism here, because the group with the lower recall completion (powder–liquid MTA) is also the group whose observed success rate would be most affected by unfavorable outcomes among non-attendees. The sensitivity analysis (Table 7) quantifies this: the magnitude of the between-group difference changes substantially under alternative assumptions about the missing outcomes, and its direction reverses between the two extreme scenarios. The complete-case estimates should therefore be interpreted as conditional on recall completion. Review of the interval records identified no documented failure and no interim assessment in any of the 22 non-assessed patients, so their outcomes are unknown rather than misclassified, and none could be retained as a documented failure.

Other limitations include the single-center design, the lack of predefined criteria for material selection, the absence of an a priori sample size calculation, the relatively short follow-up period, and the absence of a screening log before the eligibility stage, so that the number of records excluded and the reasons for exclusion cannot be reported. Because only the index tooth treated by the study operator was assessed, patients with more than one eligible molar could not be identified. Apical status was classified retrospectively from radiographs rather than recorded at treatment, and inter-examiner agreement for this classification was substantial rather than almost perfect. Because no power calculation was performed and only five failures occurred, the study may not have been able to detect clinically meaningful differences between the materials; the confidence intervals, rather than the *p*-values, describe what the data can and cannot exclude. Several details of material handling could not be verified for individual teeth: the exact mixing ratio and mixing time of the powder–liquid product, the moisture condition at placement, and whether an initial-setting check preceded liner placement; the version of the manufacturer’s instructions in use was not recorded, and the package inserts were not retained. These gaps limit the reproducibility of the protocol and prevent assessment of whether differences in application contributed to the outcomes. Because actual hemostasis times were not systematically recorded, their potential influence on treatment outcome could not be evaluated. Contemporary guidance indicates that vital pulp therapy outcomes may be more favorable when hemostasis is achieved within six minutes, so the absence of patient-level hemostasis times is a relevant limitation [5]. Similarly, because the specific pulp sensibility tests used and the duration of pain after stimulus removal were not documented in a standardized format, the original diagnostic classification could not be independently verified in detail and relied on the contemporaneous diagnosis recorded by the treating clinician. Future prospective studies should record standardized sensibility tests and the duration of provoked pain for every tooth.

Because the analyzed sample consisted exclusively of permanent molars, the generalizability of the findings to premolars and anterior permanent teeth is limited. The relatively small numbers of first and second molars also prevented meaningful analysis of treatment success according to molar type. In addition, the low number of combined failures (n = 5) precluded multivariable analysis, and classification of cavities as single-surface or multi-surface prevented separate evaluation of two- and three-surface cavities.

Radiographic assessments were independently performed by two examiners, with almost perfect inter-examiner agreement (κ = 0.880; Section 3.7). Agreement demonstrates consistency between readers; it does not show that the readings were independent of clinical information. One of the radiographic examiners had also performed the clinical recall examination and had participated in the clinical supervision of the previous treatments, and both examiners had access to the baseline radiographs during comparison. Fully independent examiners with no involvement in treatment or clinical assessment would reduce this potential observer bias in future studies. Although material allocation was concealed during the recall assessment, the possibility that previous treatment details were recalled or that material allocation could have been inferred from the radiographic appearance of the materials cannot be entirely excluded. The findings should therefore be interpreted in light of these limitations.

Clinically, these findings suggest that full pulpotomy with either formulation is feasible and may be associated with high short-term success in permanent molars treated under the specific protocol described here. They do not support conclusions about the comparative superiority, equivalence, or interchangeability of the two products, and they should not be generalized to other tooth types, other protocols, or longer follow-up. Current clinical guidance supports partial or full pulpotomy with hydraulic calcium silicate-based materials in appropriately selected permanent teeth, with intraoperative hemostasis and pulp condition informing the treatment decision [22]. In clinical practice, material selection may additionally consider handling characteristics, application time, cost, operator experience, and compatibility with the restorative protocol.

## 5. Conclusions

In this small, single-center, non-randomized observational cohort, full pulpotomy with powder–liquid MTA or premixed MTA putty from the same manufacturer was associated with high short-term combined clinical and radiographic success among the patients who completed recall (90.0% and 93.3%, respectively). No statistically significant difference was detected between the materials, and no significant association between cavity surface group and outcome was demonstrated. These findings are preliminary. Because material allocation was not randomized, loss to follow-up differed between the groups, and the confidence intervals are wide, they do not establish comparative effectiveness, equivalence, or interchangeability of the two formulations. Larger randomized clinical studies with longer follow-up, complete outcome ascertainment, and documentation of hemostasis time, root maturity, and restoration integrity are required.

## Figures and Tables

**Table 1:** Outcome definitions used in the study.

Outcome	Definition
Clinical success	Absence of all of the following at recall: spontaneous or lingering pain; tenderness to percussion or palpation; swelling or abscess; sinus tract; pathological mobility; inflammation of the surrounding soft tissues; isolated deep periodontal pocket associated with the tooth; and no root canal treatment or extraction of the tooth performed for pulpal or periapical reasons between the index pulpotomy and the recall examination.
Clinical failure	Presence of at least one of the above findings, or root canal treatment or extraction of the tooth performed for pulpal or periapical reasons between the index pulpotomy and the recall examination. Clinical status was classified before the radiographs were read; referral after the recall examination did not enter the classification.
Radiographic success	On the recall periapical radiograph, compared with the postoperative baseline radiograph: no new or enlarging periapical or furcation radiolucency and no pathological internal or external root resorption.
Radiographic failure	Presence of at least one of the above radiographic findings.
Combined success (primary outcome)	Fulfillment of both the clinical and the radiographic success criteria.
Combined failure	Failure of the clinical criteria, the radiographic criteria, or both.

Criteria adapted from Taha and Abdulkhader [3].

**Table 2:** Patient, tooth, cavity, and follow-up characteristics by material group.

Variable	Powder–Liquid MTA (n = 30)	Premixed MTA Putty (n = 30)	Total (n = 60)	*p*
Age at treatment, mean ± SD, years	11.37 ± 2.13	11.23 ± 1.94	11.30 ± 2.02	—
Age at treatment, median (range), years	11 (8–15)	11 (8–15)	11 (8–15)	0.794 ^1^
Female, n (%)	18 (60.0)	19 (63.3)	37 (61.7)	1.000 ^3^
Male, n (%)	12 (40.0)	11 (36.7)	23 (38.3)	—
First molar, n (%)	26 (86.7)	21 (70.0)	47 (78.3)	0.209 ^3^
Second molar, n (%)	4 (13.3)	9 (30.0)	13 (21.7)	—
Maxilla, n (%)	4 (13.3)	10 (33.3)	14 (23.3)	0.125 ^3^
Mandible, n (%)	26 (86.7)	20 (66.7)	46 (76.7)	—
Single-surface cavity, n (%)	7 (23.3)	9 (30.0)	16 (26.7)	0.559 ^2^
Multi-surface cavity, n (%)	23 (76.7)	21 (70.0)	44 (73.3)	—
Open apex, n (%)	5 (16.7)	5 (16.7)	10 (16.7)	1.000 ^3^
Closed apex, n (%)	25 (83.3)	25 (83.3)	50 (83.3)	—
Follow-up, median (IQR), months	11 (2)	11 (2)	11 (2)	0.907 ^1^
Follow-up, min–max, months	8–12	8–12	8–12	—

SD, standard deviation; IQR, interquartile range; min–max, minimum–maximum. ^1^ Mann–Whitney U test. ^2^ Pearson’s chi-square test. ^3^ Two-sided Fisher’s exact test.

**Table 3:** Combined clinical and radiographic outcomes by material group.

Outcome	Powder–Liquid MTA (n = 30)	Premixed MTA Putty (n = 30)	Difference, pp (95% CI)	*p*
Combined success, n (%) [95% CI]	27 (90.0) [74.4–96.5]	28 (93.3) [78.7–98.2]	+3.3 (−12.7 to +19.7)	1.000 ^1^
Combined failure, n (%)	3 (10.0)	2 (6.7)	—	—

CI, confidence interval; pp, percentage points. Confidence intervals for success rates were calculated using the Wilson score method. The absolute difference in success was calculated by subtracting the success rate of the powder–liquid MTA group from that of the premixed MTA putty group, and its 95% confidence interval was calculated using the Newcombe hybrid score method. A positive value favors premixed MTA putty. ^1^ Two-sided Fisher’s exact test.

**Table 4:** Clinical and radiographic success as separate outcomes, by material group.

Outcome	Powder–Liquid MTA (n = 30)	Premixed MTA Putty (n = 30)	Total (n = 60)	*p* ^1^
Clinical success, n (%)	28 (93.3) [78.7–98.2]	30 (100.0) [88.6–100.0]	58 (96.7) [88.6–99.1]	0.492
Clinical failure, n (%)	2 (6.7)	0 (0.0)	2 (3.3)	
Radiographic success, n (%)	27 (90.0) [74.4–96.5]	28 (93.3) [78.7–98.2]	55 (91.7) [81.9–96.4]	1.000
Radiographic failure, n (%)	3 (10.0)	2 (6.7)	5 (8.3)	
Failure on clinical criteria only, n	0	0	0	
Failure on radiographic criteria only, n	1	2	3	
Failure on both criteria, n	2	0	2	

Success rates are given with 95% confidence intervals in square brackets, calculated using the Wilson score method. ^1^ Two-sided Fisher’s exact test.

**Table 5:** Characteristics of the five teeth classified as combined failures.

Case	Material	Age/Sex	Tooth	Recall Interval, Months	Failure Finding
1	Powder–liquid MTA	11/F	46	9	Radiographic only: new periapical radiolucency
2	Powder–liquid MTA	8/F	36	11	Clinical and radiographic: tenderness to percussion, new periapical radiolucency
3	Powder–liquid MTA	14/M	46	8	Clinical and radiographic: pain, new periapical radiolucency
4	Premixed MTA putty	9/F	46	12	Radiographic only: new periapical radiolucency
5	Premixed MTA putty	15/F	37	12	Radiographic only: new periapical radiolucency

Age is given in years; F, female; M, male; tooth numbering follows the FDI two-digit notation. Apical status at baseline: open in case 2; closed in cases 1, 3, 4, and 5. Recall interval, time from pulpotomy to the recall examination; the onset of failure could not be determined. All five failures were first detected at the research recall examination and occurred in teeth with multi-surface cavities; the patients were referred for further treatment, which was not followed within the study.

**Table 6:** Combined clinical and radiographic success by cavity surface group.

Cavity Surface Group	Failure, n (%)	Success, n (%) [95% CI]	Total	*p*
Single-surface	0 (0.0)	16 (100.0) [80.6–100]	16	0.311 ^1^
Multi-surface	5 (11.4)	39 (88.6) [76.0–95.0]	44	
Total	5 (8.3)	55 (91.7) [81.9–96.4]	60	

CI, confidence interval. Confidence intervals for success rates were calculated using the Wilson score method. ^1^ Two-sided Fisher’s exact test.

**Table 7:** Sensitivity analysis of combined success under alternative assumptions about the outcomes of the 22 eligible patients who did not complete recall. The first row is the complete-case analysis (denominator: 60 assessed patients); all other rows use all 82 eligible patients as the denominator.

Assumption for Missing Outcomes	Powder–Liquid MTA, n/N (%)	Premixed MTA Putty, n/N (%)	Difference, pp (95% CI)	*p*
Complete-case analysis (assessed patients only)	27/30 (90.0)	28/30 (93.3)	+3.3 (−12.7 to 19.7)	1.000
All missing outcomes successes	44/47 (93.6)	33/35 (94.3)	+0.7 (−12.9 to 12.2)	1.000
10% of missing outcomes failures	42/47 (89.4)	32/35 (91.4)	+2.1 (−13.0 to 15.3)	1.000
20% of missing outcomes failures	41/47 (87.2)	32/35 (91.4)	+4.2 (−11.2 to 17.8)	0.726
30% of missing outcomes failures	39/47 (83.0)	31/35 (88.6)	+5.6 (−11.1 to 20.4)	0.543
50% of missing outcomes failures	35/47 (74.5)	30/35 (85.7)	+11.2 (−7.0 to 27.4)	0.276
All missing outcomes failures	27/47 (57.4)	28/35 (80.0)	+22.6 (2.0 to 39.9)	0.036
Powder–liquid missing failures; putty missing successes	27/47 (57.4)	33/35 (94.3)	+36.8 (18.5 to 51.6)	<0.001
Powder–liquid missing successes; putty missing failures	44/47 (93.6)	28/35 (80.0)	−13.6 (−30.1 to 1.1)	0.089

pp, percentage points; CI, confidence interval (Newcombe hybrid score method). The difference is the premixed putty rate minus the powder–liquid rate. Intermediate scenarios apply the stated failure proportion to the missing outcomes of both groups (17 and 5), with the resulting number of failures rounded half-up to whole teeth. Scenarios are illustrative bounds, not estimates of the outcomes of the non-assessed patients. Confidence intervals and *p*-values are conditional on the outcomes assigned in each scenario and do not account for uncertainty about those assignments. *p*, two-sided Fisher’s exact test.

## Data Availability

The anonymized data supporting the findings of this study are available from the corresponding author upon reasonable request, subject to applicable ethical and legal restrictions.

## Supplementary Materials

The following supporting information can be downloaded at: https://www.mdpi.com/article/10.3390/jcm15197563/s1. Table S1: Recorded characteristics of eligible patients according to recall-assessment status status (60 assessed, 22 not assessed); File S1: STROBE checklist.
