# Supplementary material for: Powder–Liquid Versus Premixed Putty MTA from the Same Manufacturer for Full Pulpotomy in Permanent Molars: A Short-Term Non-Randomized Observational Cohort Study
Source: J Clin Med. 2026 Sep 29;15(19):7563. doi: 10.3390/jcm15197563 (PMC13666396; doi:10.3390/jcm15197563)
Supplement: Supplementary file 1 [file jcm-15-07563-s001.zip › File S1 STROBE_checklist.pdf]

STROBE Statement — Checklist of items that should be included in reports of cohort studies

Manuscript: Powder–Liquid versus Premixed Putty MTA from the Same Manufacturer for Full Pulpotomy in Permanent Molars: A Short-Term Non-Randomized Observational Cohort Study (jcm-4569887, revised version)

Study design: single-center observational cohort study combining a retrospective review of treatment records with a prospective, standardized recall assessment with material allocation concealed from the outcome assessors (as stated in Section 2.1).

Locations refer to the numbered sections, tables and figure of the revised manuscript.

Item	Topic	Recommendation	Reported in (revised manuscript)
1a	Title and abstract	Indicate the study's design with a commonly used term in the title or the abstract	Title ("non-randomized observational cohort study"); Abstract, Methods ("single-center, non-randomized observational cohort study with material allocation concealed during outcome assessment")
1b		Provide in the abstract an informative and balanced summary of what was done and what was found	Abstract
2	Background/rationale	Explain the scientific background and rationale for the investigation being reported	Section 1, paragraphs 1–6
3	Objectives	State specific objectives, including any prespecified hypotheses	Section 1, final two paragraphs (aims; null hypotheses)
4	Study design	Present key elements of study design early in the paper	Section 2.1, paragraph 1
5	Setting	Describe the setting, locations, and relevant dates, including periods of recruitment, exposure, follow-up, and data collection	Section 2.1 (center, ethics); Section 2.2, paragraph 1 (treatment period 1 June–1 December 2025; recall 15 June–20 July 2026)
6a	Participants	Give the eligibility criteria, and the sources and methods of selection of participants; describe methods of follow-up	Section 2.2 (inclusion/exclusion criteria; record screening — no pre-eligibility screening log was kept, stated in Section 2.2); Section 2.3 (random recall order); Section 2.10 (recall assessment); Figure 1
6b		For matched studies, give matching criteria and number of exposed and unexposed	Not applicable (no matching)
7	Variables	Clearly define all outcomes, exposures, predictors, potential confounders, and effect modifiers; give diagnostic criteria, if applicable	Section 2.14 and Table 1 (outcome definitions); Section 2.9 (exposure: material; covariates); Section 2.6 (diagnostic criteria for reversible pulpitis)
8	Data sources/measurement	For each variable of interest, give sources of data and details of methods of assessment (measurement); describe comparability of assessment methods if there is more than one group	Sections 2.9 (records; retrospective two-examiner classification of apical status), 2.11 (clinical assessment), 2.12 (radiographic equipment and criteria), 2.13 (two examiners, consensus); same methods in both groups

Item	Topic	Recommendation	Reported in (revised manuscript)
9	Bias	Describe any efforts to address potential sources of bias	Section 2.3 (random recall order); Section 2.10 and 2.13 (material concealment, two independent radiographic readers; assessment sequence: coded images, random order, ≥5-day delay); Section 2.15 and 3.8 (sensitivity analysis for missing outcomes); Section 3.1 and Supplementary Table S1 (assessed vs non-assessed)
10	Study size	Explain how the study size was arrived at	Section 2.3, paragraph 2 (no a priori calculation; target of 30 per group based on available records)
11	Quantitative variables	Explain how quantitative variables were handled in the analyses; if applicable, describe which groupings were chosen and why	Section 2.9 (cavity-surface grouping; calculation of time since treatment using a common reference date); Section 2.15 (handling and analysis of age, follow-up duration, and time since treatment)
12a	Statistical methods	Describe all statistical methods, including those used to control for confounding	Section 2.15 (Fisher's exact, chi-square, Mann–Whitney U, Wilson and Newcombe intervals; no multivariable model because of five events)
12b		Describe any methods used to examine subgroups and interactions	Section 2.15 (exploratory comparison of combined success by cavity surface group; tooth type and jaw were compared only as distributions between material groups, not as outcome subgroups)
12c		Explain how missing data were addressed	Section 2.15 (complete-case primary analysis; no missing values among assessed patients)
12d		If applicable, explain how loss to follow-up was addressed	Section 2.15 (scenario-based sensitivity analysis); Section 3.8; Table 7
12e		Describe any sensitivity analyses	Section 2.15; Section 3.8; Table 7
13a	Participants	Report numbers of individuals at each stage of study—e.g., numbers potentially eligible, examined for eligibility, confirmed eligible, included in the study, completing follow-up, and analyzed	Section 3.1; Figure 1 (flow from the 82 eligible records; numbers screened before eligibility not available, stated in Sections 2.2 and 4)
13b		Give reasons for non-participation at each stage	Section 3.1 (not reachable 13; declined 4; did not attend 5); Figure 1
13c		Consider use of a flow diagram	Figure 1
14a	Descriptive data	Give characteristics of study participants (e.g., demographic, clinical, social) and information on exposures and potential confounders	Section 3.2 and Table 2 (analyzed patients by material group, including apical status); Section 3.1 and Supplementary Table S1 (assessed vs non-assessed, including time since treatment to a common reference date)

Item	Topic	Recommendation	Reported in (revised manuscript)
14b		Indicate number of participants with missing data for each variable of interest	Section 3.1 (no missing values among the 60 analyzed patients; outcomes unknown for the 22 non-assessed patients — interval records showed no documented failure and no interim assessment; Sections 2.2, 3.1)
14c		Summarise follow-up time (e.g., average and total amount)	Section 3.2 (median 11 months, IQR 2, range 8–12); Table 2
15	Outcome data	Report numbers of outcome events or summary measures over time	Sections 3.3–3.6; Tables 3–6
16a	Main results	Give unadjusted estimates and, if applicable, confounder-adjusted estimates and their precision (e.g., 95% confidence interval); make clear which confounders were adjusted for and why they were included	Sections 3.3 and 3.6; Tables 3 and 6 (unadjusted estimates with 95% CI; no adjusted estimates, reason given in Section 2.15 and Discussion)
16b		Report category boundaries when continuous variables were categorised	Not applicable; no continuous analysis variable was categorised. The 6–15-year age range was an eligibility criterion (Section 2.2).
16c		If relevant, consider translating estimates of relative risk into absolute risk for a meaningful time period	Absolute differences in percentage points with 95% CI (Tables 3, 6, 7)
17	Other analyses	Report other analyses done—e.g., analyses of subgroups and interactions, and sensitivity analyses	Sections 3.2 (tooth type, jaw), 3.4 (clinical and radiographic components), 3.5 (failed cases), 3.6 (cavity surface), 3.7 (examiner agreement), 3.8 (sensitivity analysis)
18	Key results	Summarise key results with reference to study objectives	Section 4, paragraph 1
19	Limitations	Discuss limitations of the study, taking into account sources of potential bias or imprecision; discuss both direction and magnitude of any potential bias	Section 4, limitations paragraphs (non-random allocation; differential loss to follow-up with sensitivity analysis; diagnostic verification; examiner independence; imprecision)
20	Interpretation	Give a cautious overall interpretation of results considering objectives, limitations, multiplicity of analyses, results from similar studies, and other relevant evidence	Section 4, paragraphs 1–5 and clinical-implications paragraph; Section 5
21	Generalisability	Discuss the generalisability (external validity) of the study results	Section 4, limitations (permanent molars only; single protocol) and clinical-implications paragraph
22	Funding	Give the source of funding and the role of the funders for the present study and, if applicable, for the original study on which the present article is based	Funding statement (no external funding)

An Explanation and Elaboration article discusses each checklist item and gives methodological background and published examples of transparent reporting. The STROBE checklist is best used in conjunction with this article (freely available at www.strobe-statement.org).
