# Supplementary material for: Powder–Liquid Versus Premixed Putty MTA from the Same Manufacturer for Full Pulpotomy in Permanent Molars: A Short-Term Non-Randomized Observational Cohort Study
Source: J Clin Med. 2026 Sep 29;15(19):7563. doi: 10.3390/jcm15197563 (PMC13666396; doi:10.3390/jcm15197563)
Supplement: Supplementary file 1 [file jcm-15-07563-s001.zip › Supplementary_Table_S1.pdf]

Supplementary Material

Powder–Liquid versus Premixed Putty MTA from the Same Manufacturer for Full Pulpotomy in Permanent Molars: A Short-Term Non-Randomized Observational Cohort Study

Table S1. Recorded characteristics of eligible patients according to recall-assessment status (60 assessed, 22 not assessed).

Characteristic	Assessed (n = 60)	Not assessed (n = 22)	Total (n = 82)	*p*
Age at treatment, years, mean ± SD	11.30 ± 2.02	11.05 ± 1.68	11.23 ± 1.93	0.649[1]
Age at treatment, years, median (range)	11 (8–15)	11 (8–14)	11 (8–15)	
Female, n (%)	37 (61.7)	13 (59.1)	50 (61.0)	1.000[2]
Male, n (%)	23 (38.3)	9 (40.9)	32 (39.0)	
Powder–liquid MTA, n (%)	30 (50.0)	17 (77.3)	47 (57.3)	0.043[2]
Premixed MTA putty, n (%)	30 (50.0)	5 (22.7)	35 (42.7)	
First molar, n (%)	47 (78.3)	16 (72.7)	63 (76.8)	0.571[2]
Second molar, n (%)	13 (21.7)	6 (27.3)	19 (23.2)	
Maxilla, n (%)	14 (23.3)	5 (22.7)	19 (23.2)	1.000[2]
Mandible, n (%)	46 (76.7)	17 (77.3)	63 (76.8)	
Single-surface cavity, n (%)	16 (26.7)	6 (27.3)	22 (26.8)	1.000[2]
Multi-surface cavity, n (%)	44 (73.3)	16 (72.7)	60 (73.2)	
Time since treatment to 20 July 2026, months, median (IQR)	11.5 (1.6)	11.1 (2.0)	11.5 (1.6)	0.239[1]

SD, standard deviation; IQR, interquartile range. [1] Mann–Whitney U test. [2] Two-sided Fisher's exact test. Time since treatment was calculated for all 82 patients as the interval from the treatment date to a common reference date, the end of the recall period (20 July 2026). These comparisons concern recorded characteristics only and do not exclude selection bias related to unmeasured factors.
